# Invasive ductal carcinoma arising in borderline phyllode tumor: A potential role of PIK3CA mutation

**DOI:** 10.1016/j.ijscr.2020.10.134

**Published:** 2020-11-16

**Authors:** Sarah Bouri, Philippe Simon, Nicky D’Haene, Xavier Catteau, Jean-Christophe Noël

**Affiliations:** a Department of Pathology, Erasme University Hospital, Université Libre de Bruxelles, Brussels, Belgium; b Department of Gynecology, Erasme University Hospital, Université Libre de Bruxelles, Brussels, Belgium; c Centre Universitaire Inter Regional d'Expertise en Anatomie Pathologique Hospitalière (CurePath), Jumet, Belgium

**Keywords:** Phyllode tumor, Borderline, Invasive carcinoma, PIK3CA, Mutation

## Abstract

**Introduction:**

Carcinomatous lesions associated with phyllodes tumors are extremely rare and are found in less than 1% of all cases. To date, the molecular biological mechanisms associated with this carcinomatous transformation remain unknown.

**Presentation of case:**

We present here the case of a 61-year-old patient with invasive ductal of no special type (NST) carcinoma originating in a borderline phyllode tumor with mutation in the PIK3CA gene.

**Discussion:**

To the best of our knowledge, this mutation has never been described in this type of association.

**Conclusion:**

Based on these data, we can better understand the ethiopathogenic molecular mechanisms in this type of lesion. Consequently, they could also in the future give rise to new therapeutic alternatives.

## Highlights

• Invasive carcinomatous lesions associated with borderline phyllodes tumors are extremely rare.
• Molecular biological mechanisms associated with this kind of lesions are unknown.
• PIK3CA gene mutation could be implicated in the development of these lesions.

## 1 Introduction

In European countries, phyllodes tumors represent less than 1% of all primary breast tumors [1], [2]. Among them, the benign forms represent between 35–64%, and the borderline or malignant forms 25–35% respectively [1], [2], [3]. Carcinomatous lesions associated with phyllodes tumors are extremely rare and are found in less than 1% of all cases. The majority of them being carcinoma in situ but invasive lesions have also been described in exceptional cases [4], [5]. To date, the molecular biological mechanisms associated with this carcinomatous transformation, as well as their potential therapeutic implications, remain unknown. In order to clarify these data, we tried to determine the molecular profile of a case invasive ductal of no special type (NST) carcinoma originating in a borderline phyllode tumor and occuring in a 61-year-old patient. All of these data have been reviewed in terms of their potential clinical implications. This case report has been reported in line with the SCARE criteria [6].

## 2 Case presentation

**Fig. 1:**
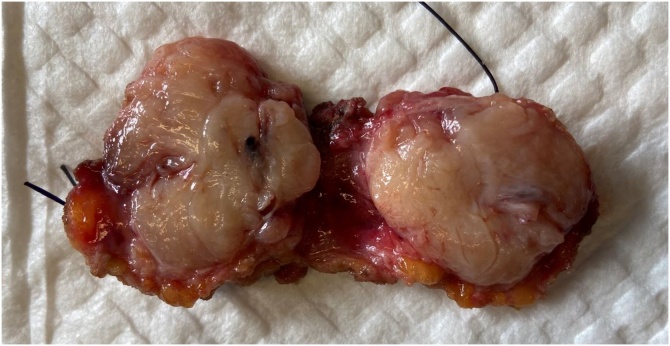
Macroscopic aspects of the tumor which appeared to be well circumscribed with nevertheless, in places, some irregular borders.

**Fig. 2:**
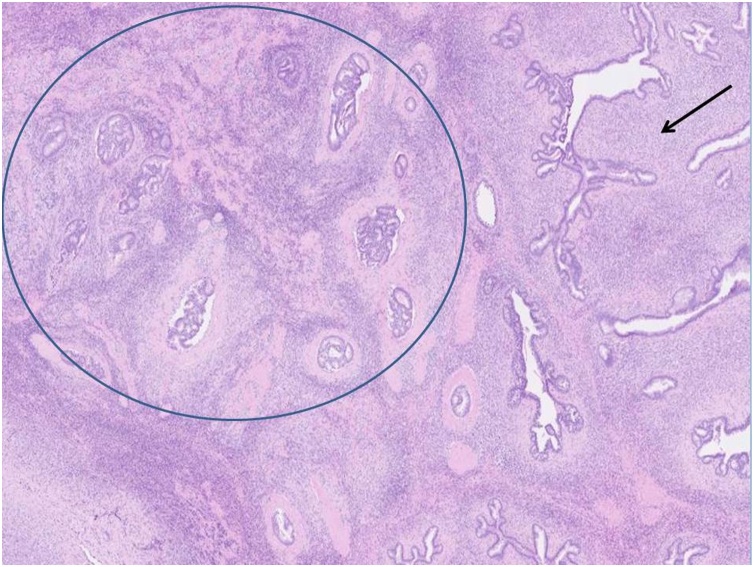
H&E staining at low power view. Grade 1 invasive carcinoma (in the circle) within the leaf-like typical aspect the phyllode tumor (arrow).

**Fig. 3:**
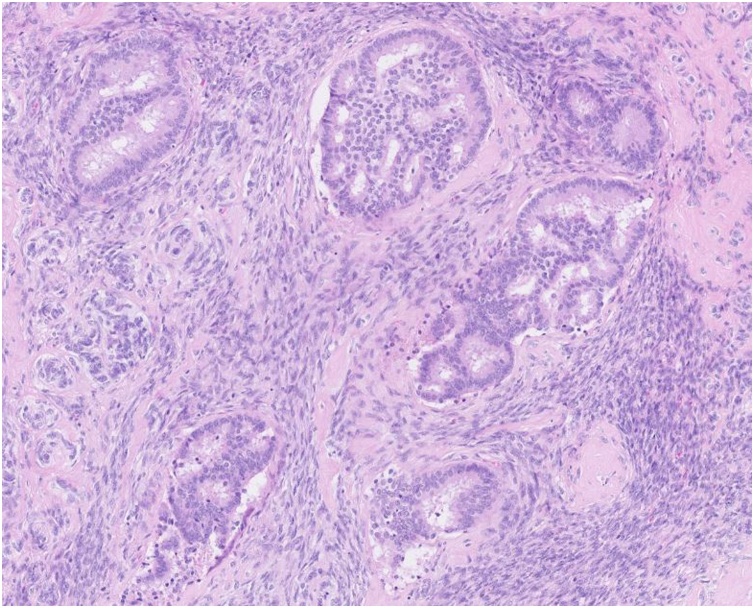
H&E staining at high power view. Grade 1 invasive carcinoma. Tumoral glands infiltrating the stroma.

**Fig. 4:**
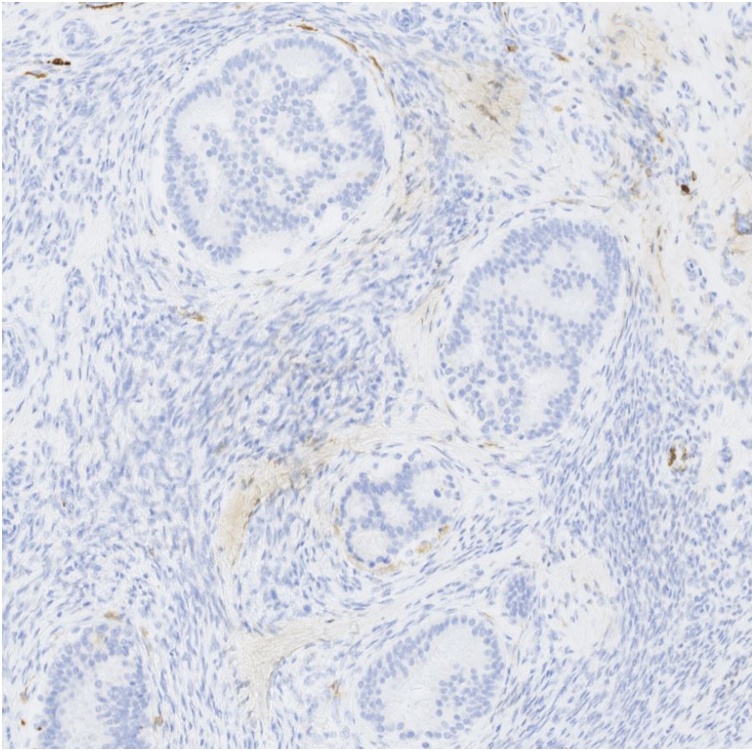
Immunohistochemistry with calponin antibody. The absence of myoepithelial cells confirms the invasive status of the tumor.

A 61 years-old female with a past history of obesity and chronic hypertension was referred to the gynecological consultation of Erasme University Hospital for a painful mass of the right breast, which have been present for 6 months according to the patient. Physical examination of the breast confirmed the presence of a retroareolar mass, indurated on palpation with a nipple invagination. The rest of the clinical examination was unremarkable. No significant drug, family or psychosocial history was noted. Radiography and ultrasonography revealed the presence of a relatively well delineated 34 × 32 mm retroareolar right breast mass without calcification classified as BIRADS 4C. There were no suspicious lymph nodes. The contralateral breast was without particularity. Biopsies of this lesion have been performed and pathological examination suggests the diagnosis of a phyllode tumor "at least borderline" with suspicion of an associated well-differentiated invasive ductal of no special type (NST) carcinoma. A lumpectomy with sentinel lymph node resection were decided after a multidisciplinary meeting and performed. Macroscopically, the tumor measures 35 mm long and demonstrates a white cut surface. It appeared to be well circumscribed with nevertheless, in places, some irregular borders (Fig. 1). Microscopically, the tumor showed prominent intracanalicular growth pattern. The stromal cellularity is non-uniform with mild or moderate atypia but without heterologous element. The stromal mitotic activity is estimated at 7 mitoses per 10 high power fields. According to these data, this phyllode tumor was classified as borderline. Within this phyllode tumor, a 3 mm focus of NST grade 1 invasive carcinoma was also found (Fig. 2, Fig. 3). It was characterized by invasive glandular structures invading the stroma without visible myoepithelial cell (p63 and calponin immunostaining were negative) (Fig. 4). By immunohistochemistry, as we have previously described, the tumoral cells were positive for hormone receptors (ER and PR), negative for HER2 and the Ki-67 index was estimated at about 5–10% [7], [8], [9]. The surgical resection margins were free and the sentinel lymph node was negative for malignancy.

After the dissection of in one part the carcinomatous component and in the other part the atypical stromal component, gene mutations testing has been performed by next generation sequencing (NGS), as we have previously validated, with a panel of 16 genes [7], [8], [9]. The E542K (exon 9) mutation of the PIK3CA gene was demonstrated both in the carcinomatous and the atypical stromal components. The surgery was well tolerated and the patient recovered after it. The adjuvant therapies included both local radiotherapy and antiestrogen tamoxifen therapy. To date, with a 3 month follow up, no recurrence or distant metastasis has occurred. Follow-up consultations every 4 months in the first two years diagnosis and yearly mammography with ultrasound have been planned.

## 3 Discussion

Invasive carcinomatous lesions associated with phyllodes tumors of the breast are exceedingly rare, with fewer than 20 cases described in the last 50 years [4], [10]. The majority of histological types being ductal invasive carcinomas/NST, invasive lobular carcinomas or squamous cell carcinomas [4], [10], [11]. These carcinomas are most often associated with benign phyllode tumors (60–80% of cases) but much more rarely with borderline or malignant ones [11], [12], [13], [14]. The molecular biological mechanisms associated with the development of these carcinomatous lesions in phyllode tumors remain poorly understood [4], [5], [10], [13]. For the first time in this work, we were able to demonstrate the presence of mutations in the phosphatidylinositol-4, 5-bisphosphate 3-kinase catalytic subunit alpha (PIK3CA) gene. The PIK3/AKT/mTOR pathway plays an important role in a number of cellular and tumoral functions, including apoptosis, proliferation, growth, survival, angiogenesis [15], [16], [17]. This pathway can be activated either directly by EGFR activation or via the RAS protein. PIK3CA mutations are present in several solid tumors and particularly in invasive NST carcinomas of the breast where they are found in about 49% of luminal A tumors [15], [16], [17], [18]. These somatic mutations are scattered in most exons, but are mainly found in the kinase and helical domains of the PIK3CA subunits with the majority mutations located on exon 9 (E542K; E545K) (as demonstrated in the present case) and exon 20 (H1047R) (18). Interestingly, PIK3CA mutations have also been implicated in the transformation of benign phyllodes tumors into borderline or malignant forms [19]. The mutational mechanisms could therefore be common to both components (carcinomatous and sarcomatous). The recurrence rate of borderline phyllode tumors varies in the literature between 14–25% but they almost never metastasize at a distance. Therefore, their classical treatment consists of excision with free margins. In the case of an invasive carcinomatous component, it seems to be useful to also include an assessment of the lymph node status. Indeed, rare cases of invasive carcinomatous tumors associated with phyllodes tumors have shown an extension to the axillary nodes [12], [20]. As both phyllode tumors and their associated carcinomas can potentially lead to distant metastases, the PIK3/mTOR inhibitors, pan-PIK3 inhibitors or the isoform-specific PI3K inhibitors which have been proved to be effective in inhibition of tumor progression could constitute future therapeutic alternatives [16], [17], [18].

## 4 Conclusion

The association of invasive carcinomatous lesions in phyllodes tumors of the breast remains exceptional. For the first time, we were able to demonstrate in this case, that a mutation in the PIK3CA gene could be implicated in the development of these lesions. These data allow us, on the one hand, to better understand these rare entities and, on the other hand, could pave the way for future alternatives therapeutic targets.

## Conflict of interest

No conflicts of interest.

## Sources of funding

No funding.

## Ethical approval

According to ULB- Erasme ethics committee, written informed consent was obtained from the patient for publication of this case report and is available for review by the Editor on request.

## Consent

According to ULB- Erasme ethics committee, written informed consent was obtained from the patient for publication of this case report and is available for review by the Editor on request.

## Author contribution

Author 1 : Sarah Bouri

-Collected the data

-Wrote the paper

Author 2 : Philippe Simon

-Contributed data or analysis tools

-Wrote the paper

-Other : Philippe Simon is also the surgeon

Author 3 : Nicky D’Haene

-Contributed data or analysis tools

-Performed the analysis

Author 4 : Xavier Catteau

-Wrote the paper

Author 5: Jean-Christophe Noël

-Conceived and designed the analysis

-Collected the data

-Wrote the paper

## Registration of research studies

N/A.

## Guarantor

Sarah Bouri MD.

## Provenance and peer review

Not commissioned, externally peer-reviewed.
